# Impacts of Hydrophobic Mismatch on Antimicrobial Peptide Efficacy and Bilayer Permeabilization

**DOI:** 10.3390/antibiotics12111624

**Published:** 2023-11-14

**Authors:** Steven Meier, Zachary M. Ridgway, Angela L. Picciano, Gregory A. Caputo

**Affiliations:** 1 Department of Chemistry & Biochemistry, Rowan University, Glassboro, NJ 08028, USAangelalpicciano@gmail.com (A.L.P.); 2 Department of Biological & Biomedical Sciences, Rowan University, Glassboro, NJ 08028, USA

**Keywords:** membrane destabilization, AMP, liposome, fluorescence spectroscopy

## Abstract

Antimicrobial resistance continues to be a major threat to world health, with the continued emergence of resistant bacterial strains. Antimicrobial peptides have emerged as an attractive option for the development of novel antimicrobial compounds in part due to their ubiquity in nature and the general lack of resistance development to this class of molecules. In this work, we analyzed the antimicrobial peptide C18G and several truncated forms for efficacy and the underlying mechanistic effects of the sequence truncation. The peptides were screened for antimicrobial efficacy against several standard laboratory strains, and further analyzed using fluorescence spectroscopy to evaluate binding to model lipid membranes and bilayer disruption. The results show a clear correlation between the length of the peptide and the antimicrobial efficacy. Furthermore, there is a correlation between peptide length and the hydrophobic thickness of the bilayer, indicating that hydrophobic mismatch is likely a contributing factor to the loss of efficacy in shorter peptides.

## 1. Introduction

The rapid development of antibiotic resistance has been recognized by the World Health Organization and the United States Centers for Disease Control (CDC) as one of the major challenges that global health faces [1]. Beyond the immediate impacts on human health, numerous NGOs and academic researchers have estimated that, by 2050, the continued growth of antimicrobial resistance may impact annual global GDPs by up to 5%, resulting in increased healthcare costs up to USD 1T per year, with more severe impacts being found in impoverished and less-developed areas of the globe [2,3].

The resistance phenomenon has been observed in numerous organisms including both Gram-positive and Gram-negative bacteria, fungi, and viruses. While methicillin-resistant *Staphylococcus aureus* (MRSA) and vancomycin-resistant enterococci (VRE) are widely known, there are many other pathogenic organisms which have been clinically isolated displaying antibiotic resistance phenotypes [4]. These developments have led to significant interest in the development of new antibiotics to combat the growing resistance phenomenon. There are numerous different approaches and classes of molecules being investigated as potential new antimicrobial treatments including traditional small molecules [5,6], peptides [7,8,9], peptide and protein mimetics [10,11,12,13,14], hydrogels [15,16], synthetic polymers [17,18], bacterial communication inhibitors [19,20], metals [21,22,23], nanoparticles [24,25], extracts from natural products [26,27], and combinatorial approaches [28,29,30].

While many different approaches are being explored for novel antimicrobials, antimicrobial peptides (AMPs) represent one of the most thoroughly studied and diverse classes of potential leads. AMPs represent a broad class of peptides with wide-ranging sources, structures, and mechanisms of action. While naturally occurring, and although AMPs are often found as components of the innate immune system (often referred to as host defense peptides), there have been many modified and synthetic variants investigated. The most well studied versions of AMPs are those which adopt an amphiphilic, α-helical secondary structure when interacting with bacterial membranes. Examples of this class of peptides include magainin, melittin, LL-37, and cecropins [31,32]. These peptides are typically short (10–30 amino acids in length), are overall net positively charged, have numerous hydrophobic groups, and often form facially amphiphilic structures when in an α-helical conformation. There is significant evidence that these peptides can provoke a disruption of the bacterial membrane; however, there are also some indications that membrane activity may only be one component of a multi-faceted mechanism of action [33,34,35,36]. Importantly, AMPs have shown a very low propensity to induce resistance development in bacteria. Despite these benefits, AMPs have found limited success in clinical applications [37].

Among the many naturally derived AMPs that have been investigated, the peptide C18G has proven to be a versatile platform for studying AMPs’ mechanism of action. The C18G sequence was originally developed as a modified extension of the C-terminal 13 amino acids of the platelet factor IV protein [38,39]. Subsequently, C18G was found to modulate signaling through several different bacterial two-component sensor systems by the disruption of protein–lipid contacts and that it may be linked to the bacterial “sensing” of AMPs [40,41,42]. Biophysical studies from our group on the C18G peptide and derivatives have demonstrated the importance of overall hydrophobic character as well as the impact of cationic amino acid side chain length on binding and antimicrobial activity [43,44,45]. These results demonstrate that C18G can cause the permeabilization of bacterial and model membranes and this activity is linked to the ability of the peptide to bind to and partition into the lipid bilayer.

The study presented here extends on the biophysical characterization of C18G with the goal of further determining the mechanism of membrane disruption. Specifically, a series of truncated peptides was created which are shorter in length than the parent C18G and thus have an overall lower hydrophobic character, lower net charge, and an overall shorter length. Herein, we show that peptide efficacy was directly tied to the length of the peptide sequence. Moreover, the shortest peptides lost the ability to disrupt model and bacterial membranes. By varying the bilayer thickness, we demonstrate that a key component of this phenomenon is linked to hydrophobic mismatch between the peptide and the bilayer.

## 2. Results

### 2.1. Peptide Composition

The amino acid sequences and selected physicochemical characteristics of the parent C18G peptide and the truncated versions are shown in Table 1. The full-length peptide, C18G-18, was modified from the original sequence by changing the amino acid at position 10 to a tryptophan residue. This change serves two purposes, the first being the incorporation of the environmentally sensitive Trp residue allowing for interrogation using fluorescence methods. The second reason for the incorporation of Trp at position 10 was to facilitate the synthesis of the series of peptides from a single precursor batch. Since solid-phase peptide synthesis proceeds from the C-terminus to the N-terminus, all three peptides would start with the same synthetic protocol, and subsequently batches of resin can be removed from the synthesis reaction to yield the truncated form while synthesis continues on the remaining resin to create the longer peptides.

The full-length and truncated peptides vary in net charge, length, and overall hydrophobicity, and all three factors are believed to play a role in the mechanism of action of many AMPs. The peptide length varies from 18 to 10 amino acids, and it has a resultant effect of varying the molecular weight of the peptides from 2216.7 Da down to 1158.5 Da, while the net charge of the peptides at pH 7 decreases from +7 to +4. The overall hydrophobic character of the peptides is also impacted because both hydrophobic and cationic residues are removed with each truncation. The grand average of hydropathicity calculates the overall hydrophobicity of a sequence, and the calculated values remain relatively similar for each of the peptides ranging from −0.354 (most hydrophobic, C18G-13) to −0.83 (least hydrophobic, C18G-10), although in the context of the range of the scale (−4.50 to +4.50), these differences may not be very significant [48]. However, using a more specialized hydrophobicity scale developed specifically for the partitioning of peptides to lipid bilayer interfaces, more significant differences are observed in the properties of the peptides [47]. Helical wheel representations of the peptides can be seen in Supplemental Figure S1 [49]. These representations show that all of the truncated versions of the peptides maintain the facial amphiphilicity which is associated with AMP activity.

### 2.2. Antimicrobial Activity

The overarching goal of this study is to help understand the core physicochemical properties that drive antimicrobial activity. Thus, the antimicrobial efficacy of the peptides was evaluated using the standard broth microdilution assay to evaluate the minimal inhibitory concentration (MIC) of the peptides. MIC values represent the lowest concentration of the compound able to prevent growth in an overnight assay. The MIC results are shown in Table 2. The results show that the placement of Trp at position 10 had minimal to no impact on antimicrobial activity. Additionally, the C18G-13 peptide exhibited mixed antimicrobial activity compared to the C18G-18, while the C18G-10 peptide lost all antimicrobial activity against the strains tested over the range of peptide concentrations tested.

### 2.3. Binding Assays

**Figure 1:**
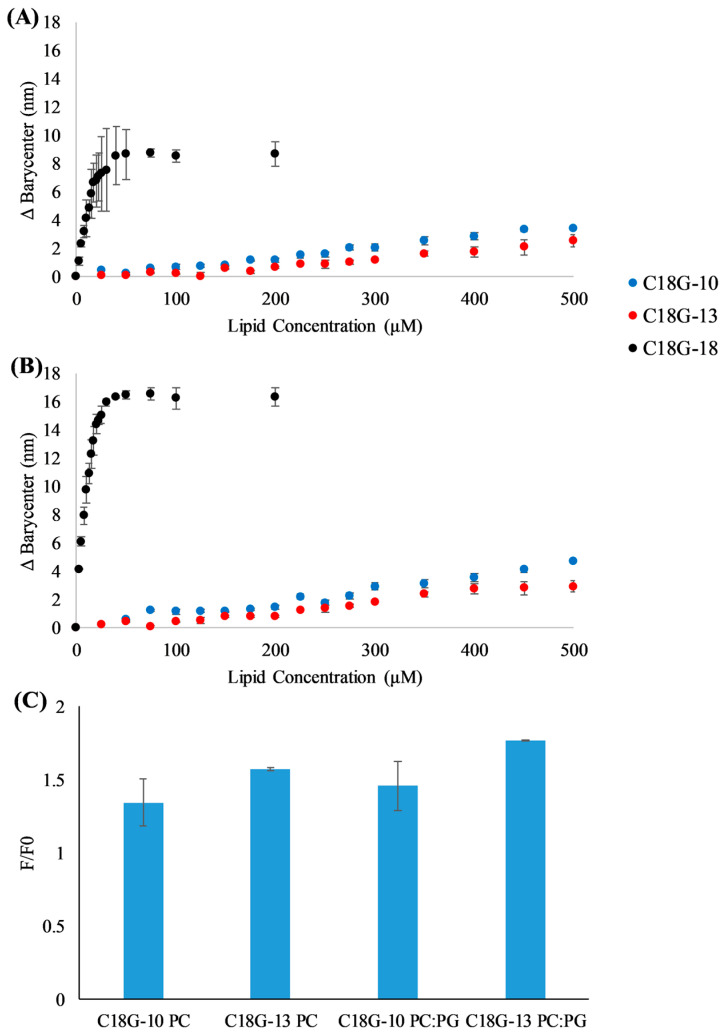
Vesicle binding assays. (**A**,**B**) Peptides C18G-18 (black), C18G-13 (red), or C18G-10 (blue) were titrated with lipid vesicles composed of (**A**) 100% DOPC or (**B**) 75% DOPC 25% DOPG. Trp fluorescence emission spectra were recorded after each addition, spectra were processed including barycenter calculation, and the final change in barycenter was calculated from the difference between the initial, 0 lipid spectra and that from each titration point. (**C**) Fluorescence intensity changes upon interaction with lipid bilayers. F/F_0_ represents the ratio of the fluorescence intensity after binding (F) compared to the intensity in the absence of vesicles (F_0_). The ratio was taken at λ = 355 nm and lipid concentration of 500 µM. All data are the averages of 2–3 independent samples and the error bars represent the standard deviations.

The first step in the activity of AMPs is the interaction with the bacterial membrane. This process is driven by both electrostatic and hydrophobic interactions, and it is complicated by the diversity of molecules presented on bacterial cell surfaces such as polysaccharides, complex lipids, and proteins. Tryptophan fluorescence emission was used to monitor peptide binding to lipid vesicles, as shown in Figure 1A,B. The binding experiments involve the addition of pre-formed lipid vesicles in which the lipid concentration is controlled to a sample containing the peptide of interest. In these experiments, we used vesicles containing 100% DOPC lipids, an approximation of mammalian or host cells, and vesicles composed of 3:1 DOPC:DOPG, an approximation of the bacterial cell membrane. Natural membranes contain significant complexity in lipid, sterol, and protein components, but these model systems are meant to replicate the two major components driving the interaction of AMPs with membranes: hydrophobicity and anionic charge. The Trp emission spectrum exhibits a blue shift when the Trp moves from a more aqueous environment (in solution) to a more non-polar environment (bound to the bilayer surface). This is analyzed by monitoring the barycenter of the emission spectrum. Consistent with previous results on C18G and other variants, the truncates in this study can interact with liposomes composed of zwitterionic lipids (Figure 1A) and a mixture of zwitterionic and anionic lipids (Figure 1B). There is a clear preference for binding to vesicles containing anionic lipids, but all peptides do interact with bilayers in the absence of the anionic lipids. Representative emission spectra can be found in Supplemental Figure S2.

As C18G-10 and C18G-13 did not exhibit significant spectral shifts, the fluorescence intensity changes upon addition of vesicles were also examined. In many cases, environmentally sensitive fluorophores will exhibit an increased fluorescence emission intensity upon binding to the lipid bilayer or other hydrophobic structures [50,51]. The emission intensity changes for C18G-13 and C18G-13, represented as F/F_0_ or the final fluorescence divided by the initial fluorescence, can be seen in Figure 1C. Notably, both peptides exhibited intensity increases upon titration with lipid vesicles 1.5–1.75-fold, indicating binding to the bilayers. Consistent with the barycenter analysis, the peptides did show a slightly enhanced fluorescence increase when interacting with anionic vesicles compared to zwitterionic vesicles, although this difference may not be significant.

### 2.4. Bacterial Membrane Permeabilization

The mechanism of action of many AMPs has been demonstrated to include bacterial membrane destabilization or disruption, including C18G. There have been numerous reported approaches to monitor membrane disruption in live bacterial cells, including measurement of membrane potential, leakage of DNA-binding dyes into the cell, and through chromogenic substrate–enzyme pairs [52].

**Figure 2:**
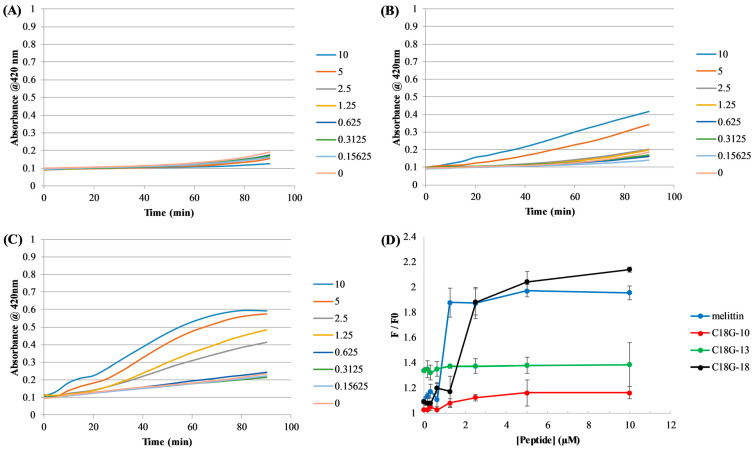
Bacterial membrane permeabilization. *E. coli* inner membrane permeabilization is shown in panels (**A**–**C**) where peptide concentrations and corresponding colors are shown in the legends on the right. Panels represent (**A**) C18G-10, (**B**) C18G-13, and (**C**) C18G-18. Data were recorded in 5 min intervals from triplicate samples and error bars represent the standard deviation. Positive controls using the detergent CTAB can be found in the supplemental information. *S. aureus* membrane permeabilization is shown in (**D**). The colors associated with each peptide and the control melittin are shown in the legend. F/F_0_ is calculated by the ratio of DAPI fluorescence at a given peptide concentration to the fluorescence prior to peptide addition. Data are from 3–5 independent samples and error bars represent the standard deviation.

The ability of the peptides to per”eabi’Ize the *E. coli* inner membrane was assessed using the cytoplasmic enzyme β-galactosidase and a chromogenic substrate ortho-Nitrophenyl-β-galactoside (ONPG). Under normal conditions, the bacterial inner membrane is relatively impermeable to the ONPG substrate; however, if it is disrupted by peptides or other molecules, the ONPG can more readily cross the membrane, resulting in an increased degree of substrate conversion. As shown in Figure 2A–C, the peptides induced varying degrees of leakage across the *E. coli* inner membrane, and all acted in a dose-dependent manner. Consistent with the MIC results, the full-length peptide was the most effective at permeabilization while C18G-10 was the least effective. Results from the control experiments using a membrane-solubilizing detergent can be found in Supplemental Figure S3.

The ability of the peptides to disrupt the membrane of Gram-positive *S. aureus* cells is shown in Figure 2D. This assay relies on the passage of the DNA-binding dye DAPI across the bacterial cell membrane. Under normal conditions, DAPI is minimally permeable across the membrane and exhibits very low to negligible fluorescence emission when in aqueous environments. Upon membrane permeabilization, the DAPI can cross the membrane and interact with cellular DNA, inducing a dramatic increase in fluorescence emission intensity. The results in Figure 2D parallel those for *E. coli*, with both peptide length and dose dependence being linked to membrane disruption. The bee venom peptide Melittin was used as a positive control.

### 2.5. Vesicle Permeabilization

In light of the results of the bacterial membrane permeabilization studies, there appears to be a link between peptide length and ability to disrupt the bilayer. In an attempt to gain more insight into this relationship, a series of dye leakage experiments were carried out in which lipid vesicles were created using lipids with varying acyl chain lengths, thus varying the thickness of the bilayer. These vesicles were created with the self-quenching dye calcein trapped in the vesicle lumen which, upon leakage from the vesicle interior, is diluted, relieves the self-quenching, and results in a large increase in fluorescence intensity. Leakage was normalized by comparing the intensity before addition to peptide as the zero value, and after the vesicles were permeabilized with the detergent Triton X-100 as the complete or 100% leakage value [53].

**Figure 3:**
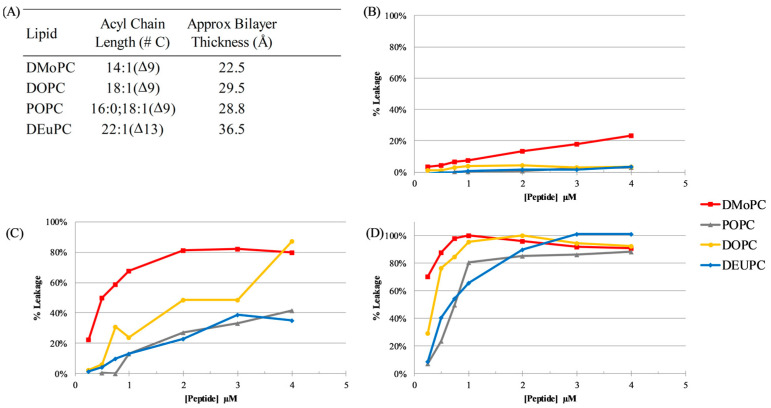
Vesicle leakage. (**A**) Synthetic lipids used to make vesicles with varied bilayer hydrophobic thickness and the approximate resultant bilayer thickness from each lipid type, based on data from references [54,55]. Leakage from vesicles of different bilayer thickness caused by (**B**) C18G-10, (**C**) C18G-13, and (**D**) C18G-18. Lipid concentration was 200 µM. Leakage percentage was determined by measuring calcein fluorescence prior to addition of peptides (zero or baseline leakage), after 60 min of incubation, and after addition of the detergent Triton X-100 (100% leakage). Data are representative samples from individual paired experiments.

Here, four different lipids were used to create vesicles: 1,2-dimyristoleoyl-sn-glycero-3-phosphocholine(14:1 (Δ9-Cis) PC; dMoPC), 1,2-dioleoyl-sn-glycero-3-phosphocholine (18:1 (Δ9-Cis) PC; DOPC), 1-palmitoyl-2-oleoyl-glycero-3-phosphocholine (16:0–18:1 PC; POPC), and 1,2-dierucoyl-sn-glycero-3-phosphocholine (22:1 (Δ13-Cis) PC; dEuPC). The acyl chain lengths and approximate resultant hydrophobic thickness of the bilayers can be found in Figure 3A, based on the measurements in references [54,55]. The results of dye leakage can be found in Figure 3B–D. These data show that a given peptide’s ability to cause membrane permeabilization is linked both to bilayer thickness and peptide length, indicating a hydrophobic matching effect. The shortest peptide, C18G-10, was able to permeabilize the thinnest bilayers tested to some extent; however, this ability was lost when moving to thicker bilayers. C18G-13 displayed significantly enhanced leakage compared to C18G-10 in all bilayer thicknesses, but still incomplete permeabilization of the thickest bilayers tested. Finally, C18G-18 was able to disrupt the vesicles of all thicknesses tested, with a nearly complete disruption of the vesicles at the highest concentrations tested. Taken together, these data indicate that hydrophobic matching of the peptide to the target bilayer is an important consideration in the mechanism of AMPs, with shorter sequences being more susceptible to losing activity.

## 3. Discussion

Hydrophobic mismatch has been an area of study for many years, primarily focused on the function of transmembrane proteins and peptides. The concept of hydrophobic matching proposes that protein transmembrane domains have evolved such that the length of the transmembrane segments “match” the hydrophobic thickness of the membrane in which they are functionally incorporated [56]. Hydrophobic matching has been linked to numerous proteins and proper function [57,58,59], sorting and location in the membrane [60,61,62,63,64], or transport across the membrane [65,66,67]. Overall, this phenomenon is important in normal cellular function for a variety of protein systems.

The effect of hydrophobic mismatch on AMPs has been investigated in some model systems, but this area is still largely unexplored. Much of the early work on hydrophobic matching with AMPs was focused on ion channel-forming peptides. These systems, such as gramicidin and alamethicin, adopt stable transmembrane orientations in order to functionally transport ions across the bilayer [4,68,69]. These experimental investigations were corroborated by simulations which showed similar mismatch dependence [70,71]. These molecules function similarly to traditional transmembrane proteins, and thus it is not surprising that they are impacted by hydrophobic mismatch. Ulrich and coworkers used model AMPs based on a repeating sequence of amino acids to investigate the role of hydrophobic mismatch by varying the number of repeats in the final sequence, and thus the length of the peptide. Using a combination of leakage experiments and NMR approaches, they demonstrated that the model AMP peptides modulated the tilt angle at which they were imbedded in the bilayer in response to hydrophobic mismatch, similar to the paradigm in transmembrane helices [72,73,74].

The results from the work presented in this paper parallel the results in Grau-Campistany et al.’s work, with decreased bilayer permeabilization in the case of negative hydrophobic mismatch (when the protein segment is shorter than the thickness of the bilayer) [74]. While the structures of these peptides were unable to be experimentally determined, three independent secondary structure prediction algorithms indicate that all three sequences are likely to adopt α-helical conformations (Supplemental Figure S4) [75,76,77]. In this conformation, the length of the helices formed by the peptides would be 15 Å, 19.5 Å, and 27 Å, respectively. The permeabilization of the vesicles is completely lost for C18G-10 when the mismatch is greater than 7.5 Å, while for C18G-18 the ability to significantly destabilize bilayers when the mismatch corresponds to ~10 Å in the dEuPC bilayers is maintained. There have been reports that some AMPs can induce modest bilayer thinning (1–2 Å); however, it is unclear how overall peptide mass would impact this phenomenon. Additionally, the peptide sequences were analyzed using the iTasser algorithm for protein structure prediction [78,79,80]. This algorithm also predicted that all three sequences would adopt helical conformations, and the best fit models are shown in Supplemental Figure S5. The helical length measurements from these models are 14.3 Å (C18G-10), 18.4 Å (C18G-13), and 26.2 Å (C18G-18), measured from the Cα of the first helical residue to the Cα of the last helical residue. Overall, the data indicate that negative mismatch (helices shorter than the hydrophobic thickness of the bilayer) is more detrimental to the AMP permeabilization of membranes compared to positive mismatch.

In the context of the AMP’s mechanism of action, the results indicate that peptide length and subsequent hydrophobic matching are important to consider for the evolution and design of membrane-disrupting AMPs. Importantly, while the mechanism of action of many AMPs involved membrane destabilization, there are several physical models by which this can occur: the barrel-stave pore model, the toroidal pore model, and the carpet model [81,82]. However, all these models involve AMPs crossing the bilayer in some sort of transmembrane architecture, either stably or transiently. While the relationship is clear between hydrophobic mismatch and stable transmembrane structures, the transient transmembrane conformations are also impacted. In the case of a peptide acting by the carpet model, a key proposed component of the mechanism is the transient membrane crossing and concomitant pore formation in the bacterial membrane. If the peptide physically cannot transit across the membrane due to any combination of physicochemical limitations, then the membrane disruption is compromised, likely leading to decreased antimicrobial activity. While these results can help guide the design of new AMPs, the key factor in activity is the interaction with bacterial membranes. Bacterial membranes are inherently more complex than the model vesicles used in this and many other studies, and they do not modulate hydrophobic thickness as dramatically as shown using synthetic lipids; thus, any matching or mismatch would be a result of changing the length of the peptide. Additionally, the inherent changes in overall peptide hydrophobicity are likely to impact the cytotoxicity of the molecules. Numerous previous studies have linked net hydrophobicity to the hemolytic activity of peptides and peptidomimetic polymers, with increased hydrophobic character resulting in increased cytotoxicity and/or hemolysis [83,84,85,86,87]. Thus, there will be a necessary interplay between sufficient length to permeabilize membranes while trying to optimize the necessary hydrophobicity to allow for membrane interaction while minimizing cytotoxic effects.

## 4. Materials and Methods

### 4.1. Materials

All chemicals and supplies were purchased from VWR (Radnor PA, USA) unless otherwise noted. The lipids 1,2-dimyristoleoyl-sn-glycero-3-phosphocholine(14:1 (Δ9-Cis) PC; dMoPC), 1,2-dioleoyl-sn-glycero-3-phosphocholine (18:1 (Δ9-Cis) PC; DOPC), 1-palmitoyl-2-oleoyl-glycero-3-phosphocholine (16:0–18:1 PC; POPC), and 1,2-dierucoyl-sn-glycero-3-phosphocholine (22:1 (Δ13-Cis) PC; dEuPC) were purchased from Avanti Polar Lipids (Alabaster AL, USA) and were stored at −20 °C as stock solutions dissolved in chloroform. All samples were measured in sodium phosphate buffer (150 mM NaCl, 50 mM NaH_2_PO_4_/Na_2_HPO_4_, pH 7.0) unless specifically indicated.

All peptides were synthesized using FMOC solid-phase synthetic methods. A rink-amide resin was used as the solid support, DMF as the primary solvent, and 20% piperidine in DMF (*v*:*v*) was used for FMOC deprotection. The removal of peptides from the rink-amide support was achieved by mixing resin with a cleavage “cocktai” of 92.5:2.5:2.5:2.5 trifluoroacetic acid (TFA):water (H_2_O):triisopropylsilane (TIPS):ethanedithiol (EDT). The cleaved peptides were isolated from the spent resin by gravity filtration through glass wool and were subsequently precipitated by dropwise addition into cold diethyl ether ((C_2_H_5_)_2_O). Reversed-phase HPLC (RP-HPLC), using a Jupiter 300 C4 column (Phenomenex, Torrance CA, USA) with the mobile phase being a linear gradient of acetonitrile and water containing 0.1% TFA, changing from 0–40% acetonitrile over 40 min, was used to purify the peptides. Confirmation of the peptide identities was performed by ESI-MS in negative ion mode.

### 4.2. Bacterial Strains and Culture

The bacterial species used were *E. coli* D31 [88], *B. subtilis* ATCC: 6633, and *S. aureus* ATCC: 27660. Beginning with a stock culture preserve at −80 °C with glycerol, the bacteria were inoculated and spread on LB–Miller agar (BD-Difco, Franklin Lakes, NJ, USA) plates. These plates were grown overnight at 37 °C to allow for the growth of single, isolated colonies. A single colony from the plates was subcultured into approximately 3 mL of fresh LB or Mueller Hinton (MH) media (BD-Difco, Franklin Lakes, NJ, USA) and grown overnight in a 37 °C shaking incubator @ ~250 rpm. After incubation, an aliquot of the overnight culture was diluted 1:200 in fresh LB or MH media and allowed to grow at 37 °C with shaking until the culture density reached an OD_600_ of approximately 0.5.

### 4.3. Minimal Inhibitory Concentration Assay

Antimicrobial activity was determined using the broth microdilution minimal inhibitory concentration (MIC) assay [89]. Briefly, cultures of bacteria were grown as described and then diluted to ~10^5^ cfu/mL in fresh MH broth. Next, 90 μL of this diluted culture was added to each well of a sterile 96-well plate containing 10 µL each of serially diluted aliquots of the peptides. The plate was covered and incubated at 37 °C for 18 h. After the 18 h incubation, optical density at 600 nm was measured using a Spectramax M5 multimode plate reader (Molecular Devices, San Jose CA, USA). Optical density readings were compared to untreated controls and sterile media to determine MIC.

### 4.4. Lipid Binding Assays

Lipid vesicles for the binding assays were created by sonication of multilamellar vesicles (MLVs). Briefly, the appropriate volumes of lipids in chloroform were dried under a gentle flow of N_2_ gas and further dried by incubation in a vacuum dessicator for at least 1 h. The resultant lipid film was rehydrated by vigorous vortexing immediately upon addition of the appropriate volume of sodium phosphate buffer to create MLVs. The MLV solution was then subjected to sonication in a high-power bath sonicator (Avanti) to produce small unilamellar vesicles (SUVs). Fluorescence experiments were performed using a JY-Horiba Fluoromax4 (JY Horiba, Edison, NJ, USA) with the emission and excitation slit widths set to 2.5 nm. The samples were prepared by mixing 2 mM peptides with the phosphate buffer and then the initial fluorescence spectrum was collected. Samples were excited at 280 nm with emission recorded over the range of 300–400 nm with 1 nm increments between measurements in the spectrum. After the spectrum was collected, the appropriate volume of lipid vesicles was added to the sample, mixed by pipetting, and allowed to incubate for 5 min at room temperature before the next measurement was taken. The spectral barycenter and Δbarycenter calculations were performed as previously described [44]. All spectra were corrected for background and dilution before barycenter calculations were performed. Data are the averages of 2–3 samples and error bars represent the standard deviations.

### 4.5. Bacterial Membrane Permeabilization

Evaluation of peptide-induced permeabilization of the *E. coli* and *S. aureus* membranes was carried out as described previously [44,45,52,90]. The permeabilization of the *E. coli* inner membrane used the chomogenic substrate ortho-Nitrophenyl-β-galactoside (ONPG), which is broken down by the cytoplasmic enzyme β-galactosidase. A sample containing *E. coli* D31 in Z-buffer 0.1 M Na_2_HPO_4_/NaH_2_PO_4_, 10 mM KCl, 1 mM MgSO_4_, 0.05 M β-mercaptoethanol, pH 7.0) T was added to a 96-well plate containing serially diluted peptides, with the cationic detergent cetyltrimethylammonium bromide (CTAB) serving as a positive control (see Supplemental Figure S3). ONPG was added immediately prior to the first measurement, and subsequent measurements were taken every 5 min for 90 min total. Data are the average of 3–5 independent samples.

The permeabilization of the *S. aureus* membrane using the membrane impermeable DNA-binding fluorophore 4′,6-diamidino-2-phenylindole (DAPI). DAPI (final concentration in 85.7 nM) was added to 100 µL of bacterial cells resuspended in HBS in a 96-well plate. Peptides were added after initial background readings stabilized. As a control, only buffer with no peptide was added to control wells. The fluorescence was recorded immediately before (time 0) and after addition (time 1), at 10, 30, and 60 min using the excitation wavelength = 358 nm and emission wavelength = 461 nm. The pore-forming peptide melittin was used as a positive control. Data are the average of 3–5 independent samples and error bars represent the standard deviations.

### 4.6. Vesicle Leakage Assays

Vesicles containing calcein were prepared as noted above, with 75mM calcein solution dissolved in HBS as the solvent. Calcein-loaded vesicles were then subjected to five rounds of freeze–thaw by alternating the sample between a liquid N_2_ bath and a 37 °C water bath. Vesicles were then extruded 21 times through a 200 nm polycarbonate filter using a syringe extruder (Avanti). Loaded vesicles were separated from untrapped calcein by passage over a G25 Sephadex column equilibrated in HBS. Fractions containing vesicles were used directly on the same day. Lipid concentration was estimated using a ratiometric method in which identical preparations containing a fluorescently labeled lipid was used to determine the dilution factor from the column separation. Final lipid concentration in the samples was 200 µM. Fluorescence measurements were taken on the Spectramax M5 plate reader using excitation 495 nm and emission 520 nm. Normalization of leakage was determined by adding 20 uL of Triton X-100 to each well, incubating in the dark for 60 min, and then remeasuring fluorescence which was used as the 100% leakage for each sample.

## 5. Conclusions

Taken together, the results from this study demonstrate that hydrophobic matching between AMPs and target membranes is an important component of membranolytic activity. The ability of AMPs to permeabilize target membranes is at the core of many AMPs’ mechanism of action, and thus critical to maintain when modifying the sequence or structure of AMPs in the development pipeline. Additionally, the relationship between AMP and membrane in hydrophobic matching may be a useful tool in the determination of mechanisms for novel and uncharacterized AMPs.

## Figures and Tables

**Table 1:** Peptide sequences and properties.

Peptide	Sequence	Length	MW	Net Charge	GRAVY ^a^	Hydrophobicity ^b^
C18G-18	ALYKKLLKKWLKSAKKLG-NH2	18	2116.7	+7	−0.45	9.58
C18G-13	LLKKWLKSAKKLG-NH2	13	1512.9	+5	−0.354	7.24
C18G-10	KWLKSAKKLG-NH2	10	1158.5	+4	−0.83	4.97

^a^—Grand average of hydropathicity calculated from reference [46], ^b^—Total hydrophobic moment calculated from reference [47]. MW = Molecular weight.

**Table 2:** Minimal inhibitory concentration (µM).

Peptide	*E. coli*	*S. aureus*	*B. subtilis*
C18G	2.5	2.5	2.5
C18G-18	5	2.5	2.5
C18G-13	5	>15	5
C18G-10	>15	>15	>15

## Data Availability

The Data are contained within the article and supplementary materials.

## Supplementary Materials

The following supporting information can be downloaded at: https://www.mdpi.com/article/10.3390/antibiotics12111624/s1, Figure S1: Helical Wheel models; Figure S2: Representative Trp emission spectra; Figure S3: Detergent control for bacterial membrane permeabilization; Figure S4: helical propensity scores; Figure S5: Structural prediction for peptides (A) C18G-10, (B) C18G-13, (C) C18G-18. Structural predictions were generated using the iTasser server and software suite which is based on homology.
