# Supplementary material for: Impacts of Hydrophobic Mismatch on Antimicrobial Peptide Efficacy and Bilayer Permeabilization
Source: Antibiotics (Basel). 2023 Nov 14;12(11):1624. doi: 10.3390/antibiotics12111624 (PMC10669323; doi:10.3390/antibiotics12111624)
Supplement: Supplementary file 1 [file antibiotics-12-01624-s001.zip › antibiotics-2707356-supplementary.pdf]

antibiotics

Supplemental Information for: Impacts of Hydrophobic Mismatch on Antimicrobial Peptide Efficacy and Bilayer Permeabilization

Steven Meier [1], Zachary M. Ridgway [1], Angela L. Picciano [1] and Gregory A. Caputo [1,2,*]

[1] Department of Chemistry & Biochemistry, Rowan University, Glassboro, NJ 08028, USA; angelalpicciano@gmail.com (A.L.P.)
[2] Department of Biological & Biomedical Sciences, Rowan University, Glassboro, NJ 08028, USA
* Correspondence: caputo@rowan.edu

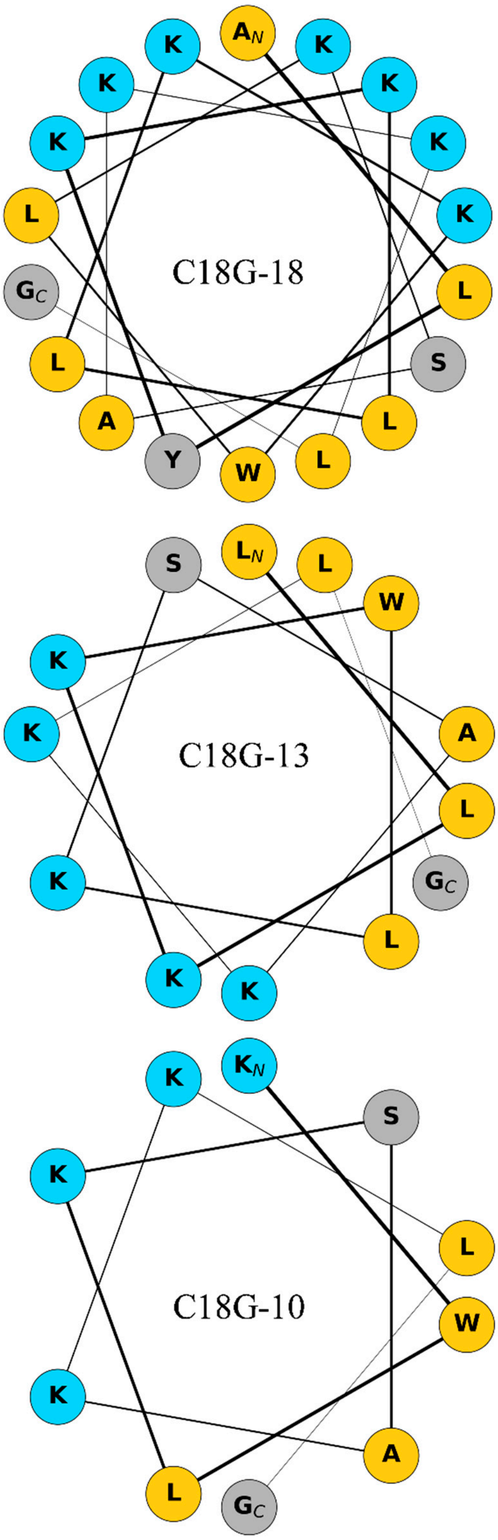

Figure S1. Helical wheel diagrams of peptides used in this study. Top—C18G-18; Middle—C18G-13; Bottom—C18G-10. Colors represent cationic (blue), hydrophobic (yellow), and polar neutral (gray) residues. Subscripts N and C refer to the N- and C-terminus of each peptide. Helical wheel diagrams made using GALAXY [49].

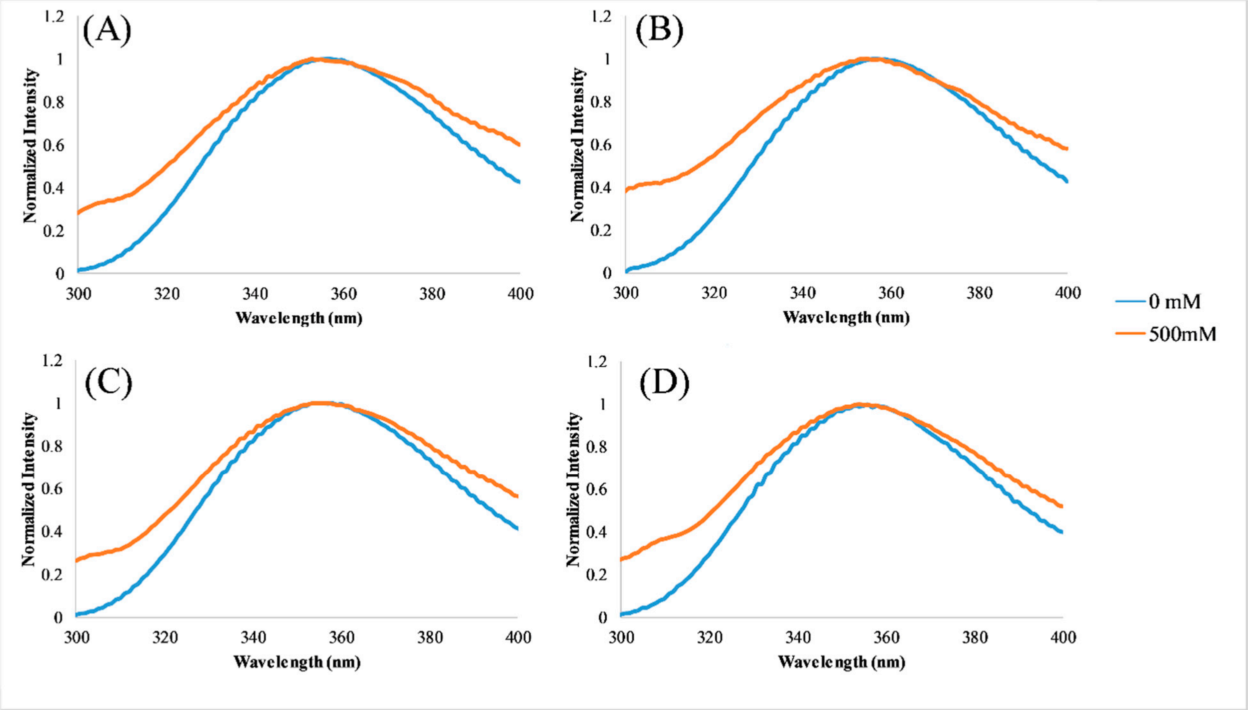

Figure S2. Representative tryptophan fluorescence emission spectra of peptides (**A**,**B**) C18G-10 and (**C**,**D**) C18G-13. Panels (**A**) and (**C**) represent samples that had 100% DOPC vesicles added, while panels (**B**) and (**D**) represent samples which had 75% DOPC 25% DOPG lipid vesicles added. Lipid concentration is shown in the legend.

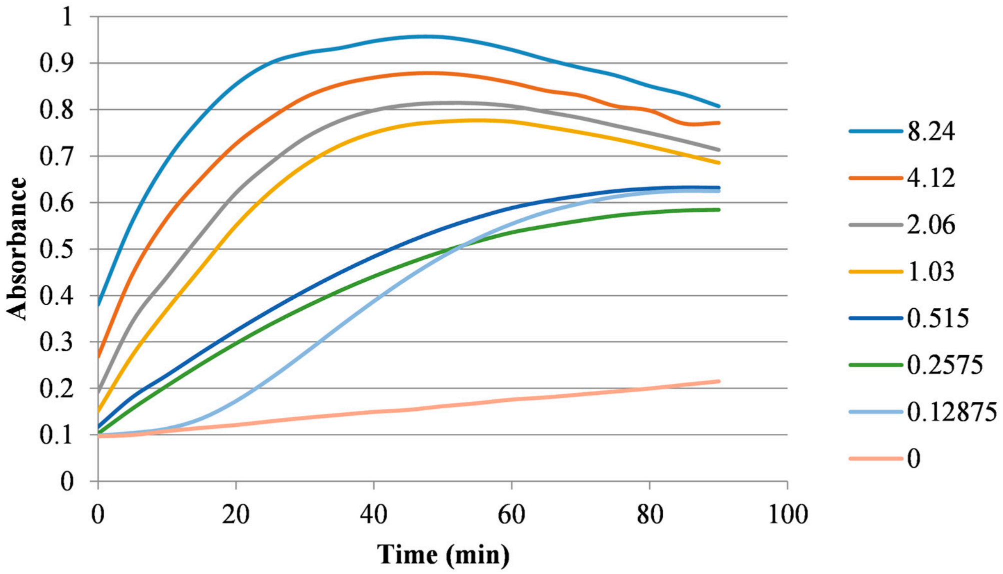

Figure S3. Permeabilization control for E. coli inner membrane permeabilization experiments. The detergent Cetyltrimethylammonium bromide (CTAB) was used as a positive control; the CTAB concentrations (in mM) and corresponding colors are shown in the legend.

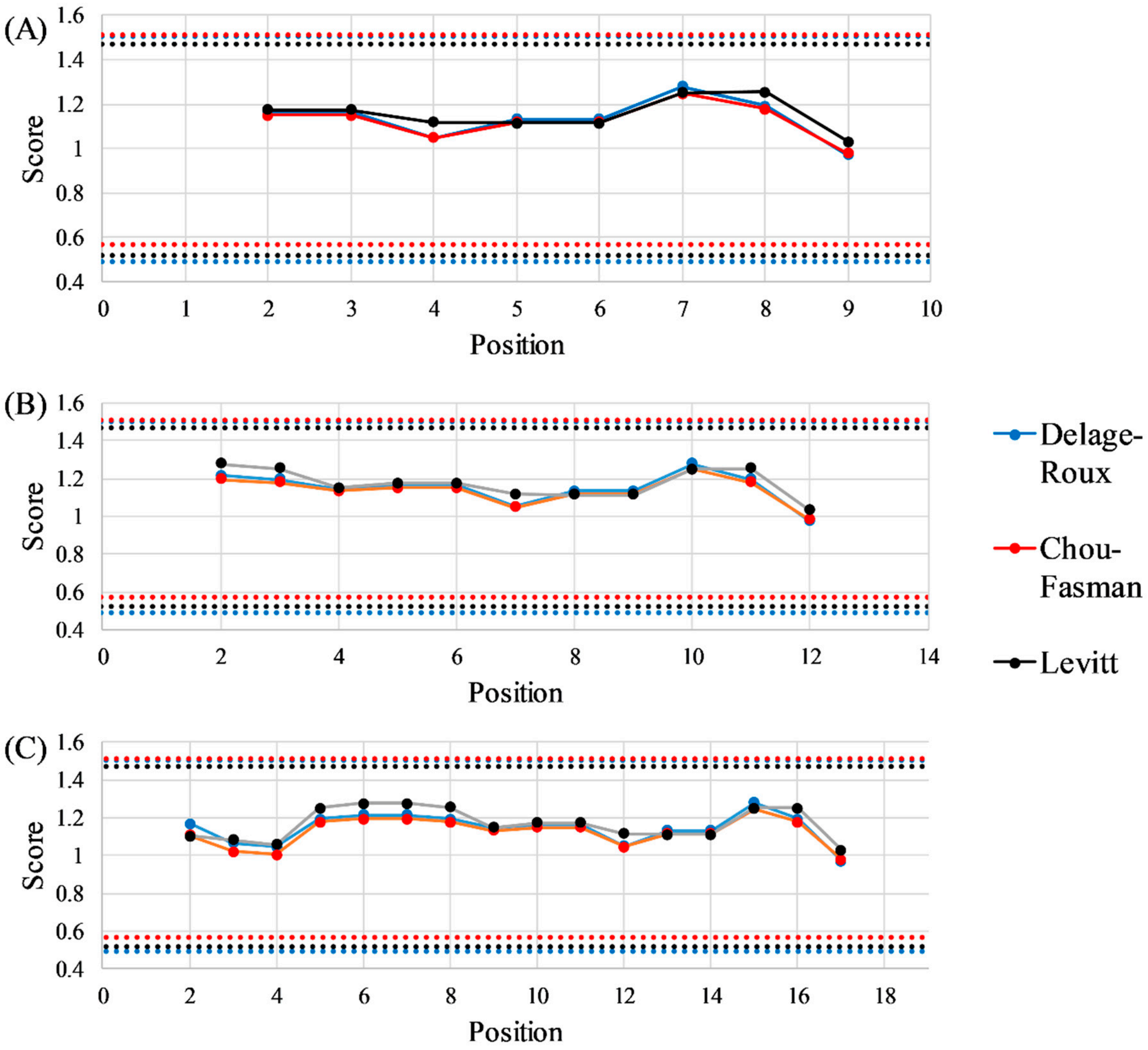

Figure S4. Helical propensity for peptides (**A**) C18G-10, (**B**) C18G-13, (**C**) C18G-18. Algorithms used and corresponding colors are denoted in the legend on the right, and the maxima and minima for each scale are represented by dotted lines of the corresponding color. Higher values indicate higher likelihood of adopting a helical conformation. Based on scales presented in references [75–77].

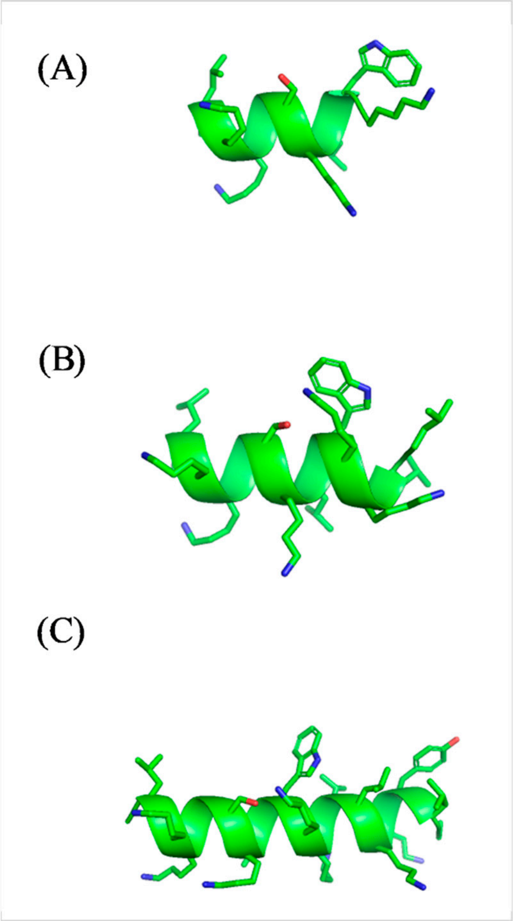

Figure S5. Structural prediction for peptides (**A**) C18G-10, (**B**) C18G-13, (**C**) C18G-18. Structural predictions were generated using the iTasser server and software suite which is based on homology [78-80]
